# Stigma and social withdrawal among colorectal cancer survivors with permanent stomas: the mediating role of social motivation in psychosocial adaptation

**DOI:** 10.3389/fpsyt.2025.1600287

**Published:** 2025-09-19

**Authors:** Qi Yao, Guopeng Li, Zhaolun Dong, Kai Liu, Xiaoling Dong

**Affiliations:** ^1^ Colorectal Surgery Ward I, Shandong Cancer Hospital and Institute, Shandong First Medical University and Shandong Academy of Medical Sciences, Jinan, China; ^2^ School of Nursing and Rehabilitation, Shandong University, Jinan, China; ^3^ Colorectal Surgery Ward II, Shandong Cancer Hospital and Institute, Shandong First Medical University and Shandong Academy of Medical Sciences, Jinan, China

**Keywords:** social withdrawal, stigma, social motivation, colorectal cancer survivors, permanent stomas

## Abstract

**Purpose:**

Social withdrawal is common among colorectal cancer (CRC) survivors with permanent stomas, and stigma can play an important role in the development of social withdrawal. However, the underlying psychological mechanisms are understudied. The current study examined the associations of stigma with social motivation, and social withdrawal among CRC survivors with permanent stomas.

**Methods:**

A cross-sectional survey was conducted with a sample of 305 CRC survivors with permanent stomas. The mediation model was conducted using the PROCESS macro for SPSS to explore the pathways through which stigma can be associated with CRC survivors’ social withdrawal, mediated by social motivation.

**Results:**

Stigma was negatively associated with social motivation (β = -0.192, P < 0.01) and positively associated with social withdrawal (β = 0.345, P < 0.001). Additionally, social motivation was negatively associated with social withdrawal (β = -0.229, P < 0.001). The mediating effect of social motivation was 0.044, accounting for 11.3% of the total effect.

**Conclusion:**

This study is the first to validate the mediating role of social motivation in this population, highlighting its significance in understanding the psychosocial adaptation of CRC survivors with permanent stomas. Future interventions aimed at preventing social withdrawal in this group should focus on tailored motivation activation strategies, particularly by actively fostering social interaction and encouraging new social connections.

## 1 Introduction

According to the 2022 global cancer statistics, colorectal cancer (CRC) accounted for approximately 1.93 million new cases, representing 9.3% of all malignant tumors, making it the third most common cancer worldwide (1). In China, an estimated 517,100 new CRC cases were reported in 2022, accounting for 10.7% of all newly diagnosed malignancies and ranking as the second most common cancer, with an overall increasing incidence trend (2). Advances in diagnostic and therapeutic techniques have significantly improved the 5-year survival rate of CRC patients. Surgical resection of the diseased intestinal segment remains the most effective curative strategy for CRC. To prevent anastomotic leakage and preserve bowel function after resection, some patients require a permanent ostomy. Notably, studies indicate that up to 37% of patients with left-sided obstructive colon cancer and 37.6% of those with rectal cancer require a permanent ostomy following curative surgery (3, 4). Although ostomy creation is a life-saving procedure, it imposes substantial physiological, psychological, and social burdens on patients (5–7). These challenges include alterations in elimination patterns and self-care routines, loss of bodily integrity, heightened emotional distress, difficulties in reintegration into family and work life, and reduced participation in social activities. Among these, social health and functioning have been identified as the most severely affected domains in CRC survivors with permanent stomas (8, 9), primarily due to the prevalent issue of social withdrawal in this population (8, 10, 11).

Social withdrawal refers to a reduction in social activities related to a target disease (12, 13), manifesting as decreased social interactions, avoidance of social engagement, reduced participation in social activities, a shrinking social network, and social isolation. Social withdrawal not only significantly limits the frequency and types of social activities but also severely restricts the maintenance and development of social relationships, leading to loneliness and social isolation and directly exacerbating impairments in social functioning (14, 15). In addition to the symptom burden and body image changes associated with cancer and its treatment, the ostomy itself presents challenges such as uncontrollable gas, odor, leakage, and the need for ongoing care. To avoid embarrassment, anxiety, or potential rejection, CRC survivors with permanent stomas often choose to conceal or selectively avoid disclosing their health condition rather than openly discussing it. Consequently, during their return to work and social life, many CRC survivors with permanent stomas opt for avoidance or withdrawal in response to various physical and psychological challenges, ultimately exhibiting social withdrawal behaviors (16). Qualitative studies have identified social engagement and participation in social activities as among the most prevalent and challenging concerns for CRC survivors with permanent stomas postoperatively (17). Survey-based studies further indicate that CRC survivors with permanent stomas often report lower rates of return to work and higher levels of social avoidance, additionally, 30% to 50% experience a significant decline in social willingness and activity following ostomy surgery, with 11.9% reporting social rejection from others (18, 19). Given the profound impact of social withdrawal and the need to develop effective intervention strategies, the first crucial step is to identify key factors associated with social withdrawal among CRC survivors with permanent stomas.

According to the cognitive integration model of internalized stigma (20), social withdrawal is seen as a behavioral manifestation of internalized stigma. The model suggests that individuals with stigma tend to adopt avoidant coping strategies (e.g., social avoidance, social withdrawal) to avoid stimuli that might trigger feelings of stigma. While this avoidance may provide short-term relief from emotional distress, over time, such behaviors reinforce the internalization of stigma and strengthen individuals’ identification with the shameful label. Furthermore, the avoidance of negative emotional experiences deprives patients of opportunities to cope and adjust, exacerbating withdrawal, isolation, and loneliness, thus creating a vicious cycle. Stigma primarily encompasses internal feelings of shame and external experiences of discrimination, often described as an indicator of social disgrace (21, 22). This can lead to emotional distress, self-deprecation, and a decline in well-being (23, 24). Due to the loss of bodily integrity, changes in elimination patterns, and reduced sense of control associated with ostomy, patients often experience feelings of inferiority, shame, and alienation, resulting in widespread internalized stigma (25–27). Reports indicate that over 44% of CRC survivors with permanent stomas experience moderate to severe internalized stigma (25–27). Social withdrawal is a transdiagnostic symptom. Empirical studies on individuals with mental illnesses, who exhibit typical social withdrawal behaviors, have identified stigma as a primary driver of social withdrawal in these populations, as they seek to avoid potential rejection or discriminatory situations (13, 28, 29). Similarly, several qualitative studies on CRC survivors with permanent stomas have found that concerns about embarrassment or potential rejection in social situations lead to a reduction or refusal to engage in social activities, highlighting the significant role of internalized stigma in social withdrawal among this group (8, 10, 11). However, there is a lack of quantitative research to validate this relationship.

The social motivation theory posits that social motivation serves as a core regulatory mechanism of social needs, referring to the psychological tendencies and biological mechanisms that drive individuals to initiate social orientation, social exploration and preference, and social maintenance (30). The behavioral characteristics driven by social motivation primarily manifest in three aspects: an innate sensitivity to social stimuli; social activities being inherently rewarding and reinforcing; and a willingness to engage in sustained social interactions. Establishing and maintaining social interactions are key manifestations of social motivation, encompassing behaviors aimed at forming, sustaining, and enhancing relationships with others. This theory suggests that social motivation is a fundamental driving force behind human social behavior, and abnormalities in motivation are considered central mechanisms underlying social behavior disorders (30, 31). Animal studies have demonstrated that inflammatory stimuli not only impair the central nervous system’s regulation of social motivation but also induce behaviors such as withdrawal, social avoidance, or aggression (32, 33). Human studies have also reported that individuals with lower self-reported social motivation exhibit greater avoidance behaviors in approach-avoidance experimental tasks and display reduced positive social emotional responses (34, 35). Additionally, the “why try” effect model of stigma suggests that stigma reduces individuals’ pursuit of valued goals, primarily through a series of key psychosocial mediators, including self-esteem and self-efficacy (36). Notably, these mediating factors, such as self-efficacy, are rooted in individuals’ intrinsic motivation systems, directly influencing behavior choices, effort, perseverance, achievement, and self-regulation (37). This indicates that reduced motivation may play a crucial role in stigma-related goal disengagement or experiential avoidance (e.g., social withdrawal). Investigating the role of social motivation in the relationship between stigma and social withdrawal may provide empirical evidence for elucidating the psychological mechanisms underlying stigma associated social withdrawal.

However, limited research has examined the mechanisms linking stigma and social withdrawal among CRC survivors with permanent stomas. Understanding these mechanisms is clinically important, as it can provide evidence to develop psychosocial interventions aimed at enhancing social motivation and reducing social isolation in this vulnerable population. Thus, this study was conducted to examine whether stigma is associated with social withdrawal among CRC survivors with permanent stomas, and whether social motivation can play a mediating role in the relationship between them. It was hypothesized that social motivation could play a mediating role in the relationship between stigma and social withdrawal.

## 2 Methods

### 2.1 Design and participants

A cross-sectional survey study was conducted at the ostomy outpatient clinic in Shandong Cancer Hospital and Institute between May 2024 and January 2025. This study was approved by the Ethics Committee of Shandong Cancer Hospital and Institute (Approval No. SDTHEC 2023001016). All CRC survivors with permanent stomas who provided oral informed consent were enrolled in this study. The inclusion criteria were as follows: (1) age 18 or older; (2) diagnosed with CRC and treated with permanent colostomy; (3) received surgery at least one month before the study; and (4) able to understand and answer the questionnaires. The exclusion criteria were as follows: (1) CRC survivors who had a reversed temporary ostomy; and (2) severe mental illness or cognitive impairment that would impede their ability to complete the survey. The minimum sample size was estimated as 204 by G*Power software for multiple regression using the following parameters (38): medium effect size of 0.15, alpha of 0.05, and power of 0.95, test predictors of 16 (i.e., age, gender, marital status, residence, education, employment, monthly income, BMI, time since operation, stoma location, current treatment status, stoma-related problem, independence of stoma self-care, complication or not, stigma, and social motivation). A convenience sample of 320 participants who meet the inclusion criteria were invited to take part in this study. Among them, 12 declined participations for a variety of reasons, including lack of interest and time conflicts, 3 provided incomplete data, and finally 305 participants completed all the questionnaires and included in the analysis.

### 2.2 Measures

A self-administered, structured questionnaire including a study-specific questionnaire on socio-demographic variables and clinically relevant variables, stigma, social motivation, and social withdrawal, was used to collect data.

#### 2.2.1 Socio-demographic and clinical variables

The demographic and clinical questionnaire was developed to elicit information including participants’ age, gender, marital status, residence, education, employment, monthly income, BMI, time since operation, stoma location, current treatment status, stoma-related problem, independence of stoma self-care, and complication or not.

#### 2.2.2 Stigma

The self-designed stigma scale comprises two items that assesses the extent of perceived stigma by asking about shame and discrimination resulting from having a permanent colostomy. Shame and discrimination were thought to be the key elements of stigma (14, 21) and designed for assessing stigma in this study. The items are as follows: ‘Because of your illness or stoma, have you ever felt: (1) shame; (2) discrimination.’ The items are rated on an 8-point scale that ranges from ‘0 = I have not felt this’ to ‘7 = I have always felt this’. The raw total scores range from 0 to 14, with higher scores indicate higher stigma experience. This two-items stigma scale has demonstrated good reliability in infertility condition (39). In the present study, Cronbach’s alpha for the two-items stigma scale was 0.839.

#### 2.2.3 Social motivation

The state motivation to foster social connection scale was used to assess self-reported motivation to engage in social connections with existing and with new social targets among CRC survivors with permanent stomas (40). The 10-item scale is comprised of two 5-item subscales: state motivation to foster social connection with new (SMSC-N) (e.g., “Right now, I would like to meet new people”) and state motivation to foster social connection with existing (SMSC-E) (e.g., “Right now, I’d like to be around friends”), measured from 1 (strongly disagree) to 5 (strongly agree) for each item. The sum of all item scores is the total score in each subscale, with higher values indicating a greater desire to foster social connections with others. In the present study, Cronbach’s alpha for the total scale and its two domains were 0.866, 0.846 and 0.891, respectively,

#### 2.2.4 Social withdrawal

The social withdrawal subscale (SWS) from internalized stigma for mental illness scale (ISMI) was used to assess the extent of perceived social withdrawal among CRC survivors with permanent stomas (41). The ISMI scale was originally developed by Boyd et al. (41), and the Chinese version was translated and developed by Li et al. (42). The ISMI scale is a 29-item questionnaire designed to measure self-stigma among persons with psychiatric disorders, producing five subscales: alienation, stereotype endorsement, discrimination experience, social withdrawal, and stigma resistance. Of these, the SWS contains six items. In this study, the original term ‘mental illness” was replaced with “stoma”, such as ‘I don’t socialize as much as I used to because my *stoma* might make me look or behave weird’ and ‘I avoid getting close to people who don’t have *stoma* to avoid rejection.’ The items are rated on a 4-point scale that ranges from ‘1 = strongly disagree’ to ‘4 = strongly agree’. The sum of all items scores is the total score, with high values indicating a higher self-perceived social withdrawal. Existing research has confirmed that the SWS demonstrates acceptable reliability and validity for measuring social withdrawal among CRC survivors with permanent stomas (16). In the present study, Cronbach’s alpha for the SWS was 0.864.

### 2.3 Statistical analyses

The data analysis was conducted by IBM SPSS 27.0 (IBM Corp., 2020). Mean ± standard deviations (after confirmation of normal distribution using the Kolmogorov–Smirnov test), frequency, and percentages were used to describe the characteristics of the participants. Independent t-test and analysis of variance (ANOVA) were used to examine the differences in social withdrawal by sample characteristics. Pearson’s correlations were used to examine the associations among stigma, social motivation, and social withdrawal. Before conducting parametric analyses (independent t-test, ANOVA, and Pearson correlations), assumptions of normality, homogeneity of variance, and linearity were examined and reasonably met.

Finally, the hypothesized mediation model was tested by using the SPSS PROCESS 4.0 macro software, which was specifically developed for testing the complex models (43). In PROCESS, model 4 was applied for mediation analysis. The indirect effect was estimated by implementing the bootstrap technique with 5000 bias-corrected bootstrap samples. If the 95% confidence interval (CI) did not include 0, the mediation effect was considered statistically significant. The covariates with a P value lower than 0.1 in the univariate analysis were included in the mediation model. All the predictors were standardized to avoid multicollinearity. P values reported were two tailed, and P value < 0.05 was considered significant.

## 3 Results

### 3.1 Socio-demographic, clinical characteristics, and univariate analyses of social withdrawal

The characteristics of the patients are summarized in **Table 1**. Of the 305 patients included, the mean age of patients was 59.55 (SD = 11.08) years, 60.3% of patients were male, 93.4% were married, 49.8% lived in urban, 59.0% had less than high school, 38.0% were employed, and 32.1% had a low monthly income. The mean BMI was 23.69 (SD = 3.65) kg/m^2^, 56.7% of patients were normal weight (BMI ranging from 18.5 to 24.9). Regarding clinical information, the mean time since ostomy was 8.61 (SD = 7.65, range = 1 – 34) months. 19.3% of patients received surgery more than 12 months, 78.7% of patients with the stoma located on the left of the abdominal wall, 43.9% were undergoing postoperative adjuvant therapy, 31.1% had complications related to the enterostomy, 49.8% of patients were unable to achieve independence in stoma self-care, and 32.8% had complications related to chronic disease. The mean score of social withdrawal was 15.18 (SD = 2.98). The independent t-test, ANOVA and Pearson correlation results were reported in **Table 1**. The scores of social withdrawal were not significantly different with socio-demographic and clinical data.

**Table 1:** Univariate associations of socio-demographic and clinical characteristics with social withdrawal (n = 305).

Variables	Range/group	Total N (%)/M ± SD	Social withdrawal (M ± SD)	*T/f/r*	P
Age (years)	23 - 80	59.75 ± 11.08	15.18 ± 2.98	*r* = -0.047	0.414
Gender	Male	184 (60.3)	14.91 ± 2.59	*t* = 1.843	0.067
	Female	121 (39.7)	15.59 ± 3.47		
Marital status	Married	285 (93.4)	15.12 ± 3.01	*t* = 1.200	0.231
	Other	20 (6.6)	15.95 ± 2.52		
Residence	Urban	152 (49.8)	15.26 ± 2.99	*t* = 0.502	0.616
	Rural	153 (50.2)	15.09 ± 2.98		
Education	Less than high school	180 (59.0)	15.33 ± 3.03	*F* = 0.663	0.516
	High school	66 (21.6)	14.86 ± 3.11		
	College or higher	59 (19.3)	15.05 ± 2.69		
Employment	Employed	116 (38.0)	15.16 ± 2.97	*t* = 0.100	0.920
	Unemployed	189 (62.0)	15.19 ± 3.00		
Monthly income (¥)	< 3000	98 (32.1)	15.13 ± 3.47	*F* = 0.142	0.868
	3000 – 6000	162 (53.1)	15.25 ± 2.74		
	> 6000	45 (14.8)	15.00 ± 2.72		
BMI (kg/m2)	14.53 – 33.48	23.69 ± 3.65		*F* = 0.068	0.934
	< 18.5	23 (7.5)	15.35 ± 3.71		
	18.5-24.9	173 (56.7)	15.20 ± 2.85		
	> 24.9	109 (35.7)	15.11 ± 3.05		
Time since operation (months)	1 - 34	8.61 ± 7.65		*F* = 1.331	0.264
	1 - 3	114 (37.4)	15.29 ± 3.27		
	4 - 6	73 (23.9)	14.59 ± 3.06		
	7-12	59 (19.3)	15.53 ± 2.72		
	> 12	59 (19.3)	15.34 ± 2.48		
Stoma location	Left	240 (78.7)	15.12 ± 2.98	*t* = 0.632	0.528
	Right	65 (21.3)	15.38 ± 3.01		
Current treatment	Under	134 (43.9)	14.82 ± 2.98	*t* = 1.815	0.071
	No or finished	171 (56.1)	15.45 ± 2.96		
Stoma-related problem	Yes	95 (31.1)	15.25 ± 3.26	*t* = 0.297	0.766
	No	210 (68.9)	15.14 ± 2.86		
Independence of	Fully	35 (11.5)	15.60 ± 2.02	*F* = 0.549	0.578
stoma self-care	Partially	118 (38.7)	15.01 ± 2.92		
	Uncontrollably	152(49.8)	15.21 ± 3.21		
Complication or not	Yes	100 (32.8)	15.17 ± 3.15	*t* = 0.100	0.921
	No	205 (67.2)	15.20 ± 2.62		

M, mean; SD, Standard Deviation.

### 3.2 Common method bias

Harman’s single-factor test was used to identify common method bias. The results showed that there was a total of 4 factors with eigenvalues were greater than 1. The loading of the first factor was 28.5%, less than 40% of the covariance among all the items. This suggested that there was no significant common method bias in this study.

### 3.3 Correlational analyses

Results from Pearson correlation analyses were presented in **Table 2** and revealed that social withdrawal was positively associated with stigma (*r* = 0.400, *P* < 0.001), and negatively associated with social motivation (*r* = -0.300, *P* < 0.001). Besides, stigma was negatively associated with social motivation (*r* = -0.205, *P* < 0.001).

**Table 2:** Correlations, means, and standard deviations of study variables (n = 305).

Variables	M ± SD	Range	1	1.1	1.2	2
1. Social withdrawal	15.18 ± 2.98	6 - 24	1			
2. Stigma	5.52 ± 3.64	0 - 14	0.400***	1		
3. Social motivation	33.99 ± 6.34	13 - 50	-0.300***	-0.205***	1	
3.1 SMSC-N	14.99 ± 4.08	5 - 25	-0.333***	-0.195***	0.845***	1
3.2 SMSC-E	19.00 ± 3.63	8 - 25	-0.149***	-0.139*	0.798***	0.353***

M, mean; SD, Standard Deviation. SMSC-N state motivation to foster social connection with new, SMSC-E state motivation to foster social connection with existing.

*P < 0.05, **P < 0.01, ***P < 0.001.

### 3.4 Mediation analyses

**Figure 1:**
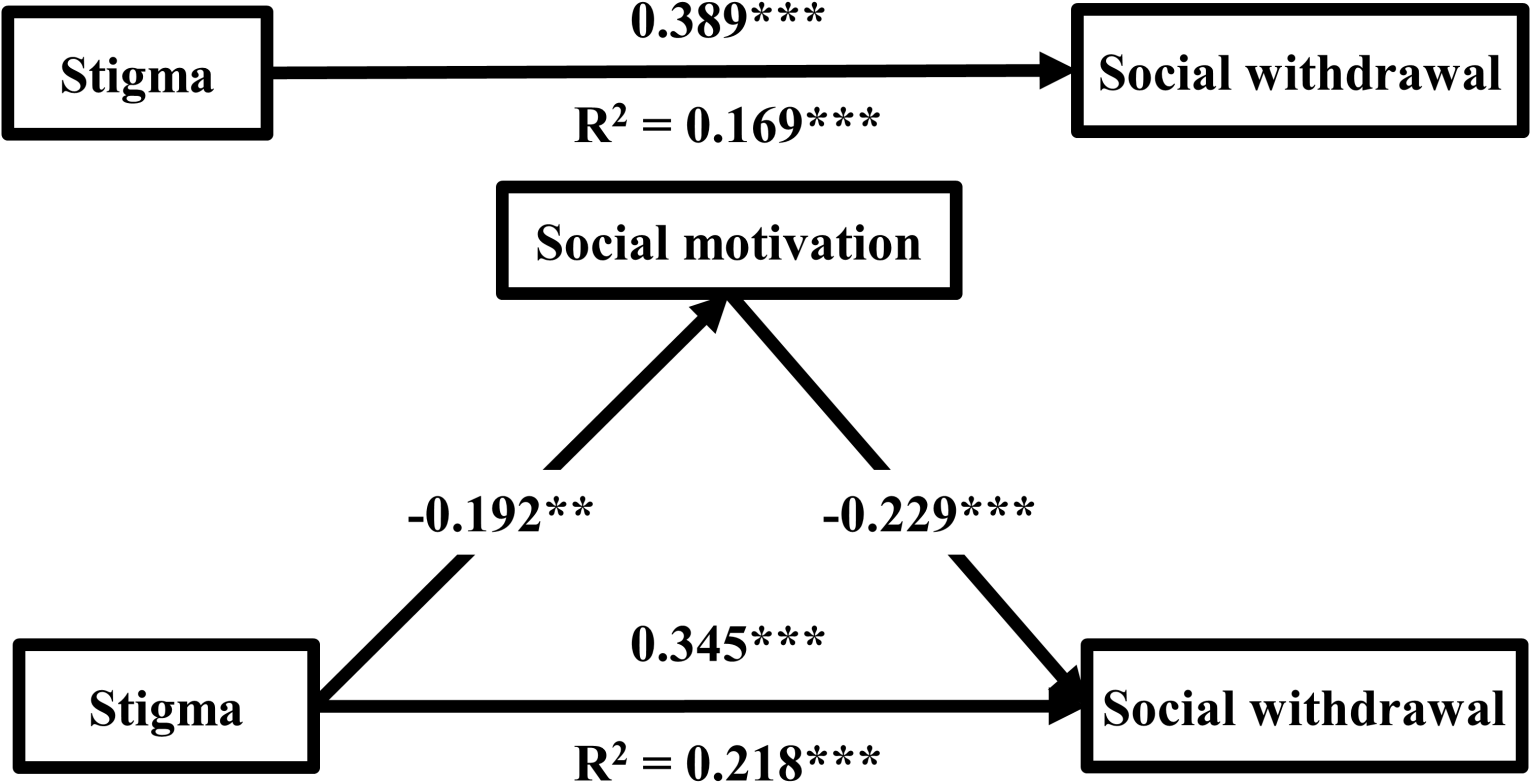
The mediating models of social motivation between stigma and social withdrawal (n = 305). Gender, and current treatment status were adjusted as covariates in all analyses. *P < 0.05, **P < 0.01, ***P < 0.001.

The mediation model examined whether social motivation mediated the association between stigma and social withdrawal. The results, generated using PROCESS macro model 4, were presented in **Table 3** and **Figure 1**. After adjusting for gender and current treatment status, the mediation analysis revealed that stigma was negatively associated with social motivation (*β* = -0.192, *P* < 0.01) and positively associated with social withdrawal (*β* = 0.345, *P* < 0.001). Additionally, social motivation was negatively associated with social withdrawal (*β* = -0.229, *P* < 0.001). The mediating effect of social motivation was 0.044 (-0.192 × -0.229), accounting for 11.3% of the total effect (0.044/0.389). Bootstrapping results showed that the 95% CI for the mediation effect ranged from 0.017 to 0.080, excluding 0, indicating that social motivation partially mediated the relationship between stigma and social withdrawal.

**Table 3:** The mediating effects of social motivation and its two domains between stigma and social withdrawal (n = 305).

Pathways	Effect value	SE	95% CI		Effect size
			Lower	Upper	
Stigma → Withdrawal (Total effect)	0.389	0.053	**0.285**	**0.494**	100%
Stigma → Withdrawal (Direct effect)	0.345	0.053	**0.242**	**0.449**	88.7%
Stigma → Motivation → Withdrawal (Indirect effect)	0.044	0.016	**0.017**	**0.080**	11.3%
Stigma → Withdrawal (Total direct effect)	0.048	0.018	**0.017**	**0.086**	12.3%
Stigma → SMSC-N → Withdrawal (Indirect effect 1)	0.046	0.017	**0.016**	**0.084**	11.8%
Stigma → SMSC-E → Withdrawal (Indirect effect 2)	0.002	0.008	-0.013	0.019	0.5%
Indirect effect 1 – Indirect effect 2	0.045	0.020	**0.008**	**0.088**	

SE, standard error; CI, confidence interval.

Bold values, the 95% confidence interval (CI) does not include 0.

**Figure 2:**
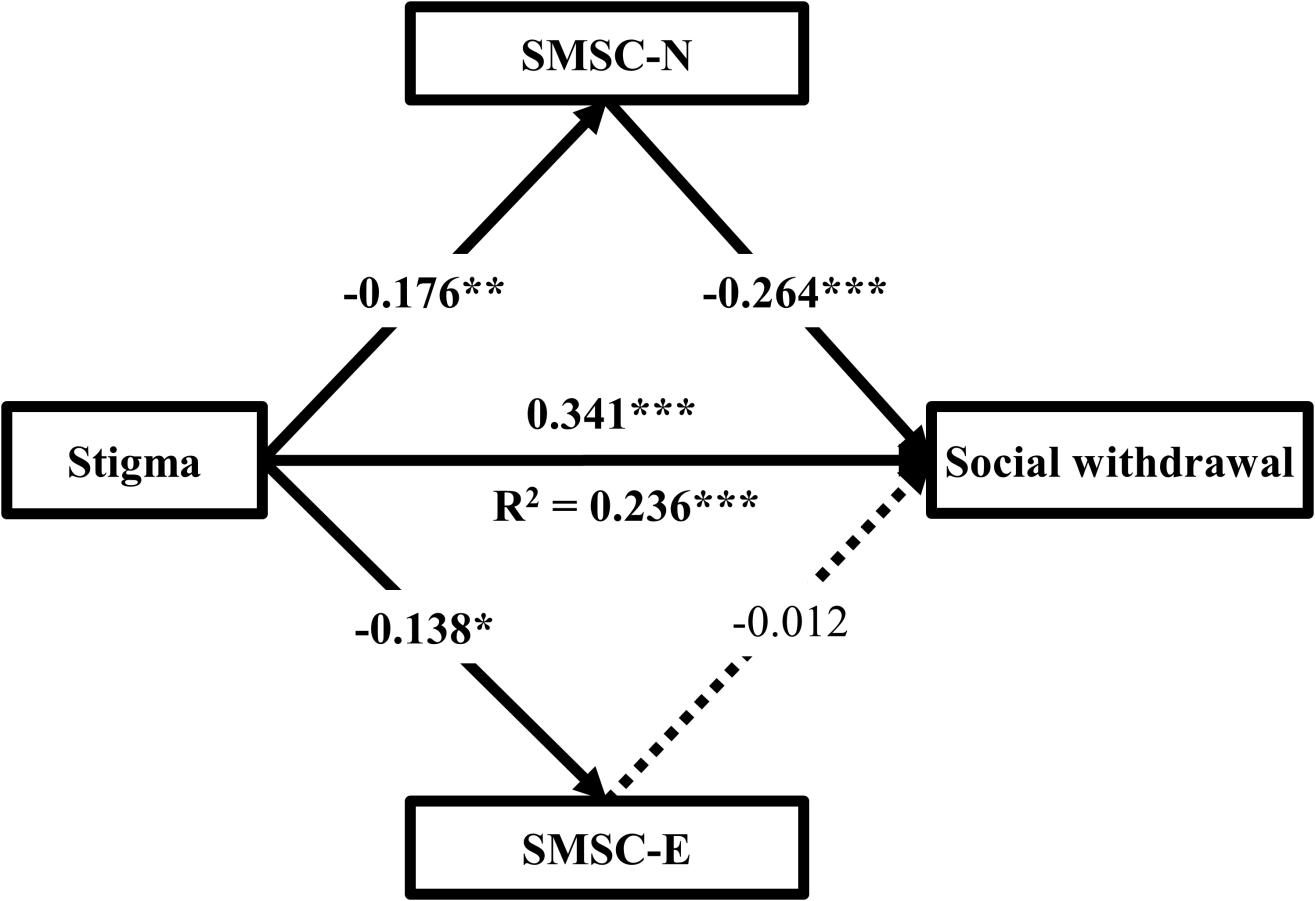
The mediating models of two domains of social motivation between stigma and social withdrawal (n = 305). Gender, and current treatment status were adjusted as covariates in all analyses. SMSC-N state motivation to foster social connection with new, SMSC-E state motivation to foster social connection with existing. *P < 0.05, **P < 0.01, ***P < 0.001.

Considering the fundamental differences between the social motivation for new and for existing (40), another mediation model established to further explore whether their roles in the relationship between stigma and social withdrawal differed. The results were presented in **Table 3** and **Figure 2**. The mediating effect of social motivation for new was 0.046 (-0.176 × -0.264), accounting for 11.8% of the total effect (0.046/0.389). Bootstrapping results showed that the 95% CI for the mediation effect ranged from 0.016 to 0.084, excluding 0, indicating that social motivation for new partially mediated the relationship between stigma and social withdrawal. In contrast, Bootstrapping results showed that the 95% CI for the mediation effect of social motivation for existing ranged from -0.013 to 0.019, including 0, indicating that social motivation for existing did not mediated the relationship between stigma and social withdrawal. The contrast result between the two indirect pathways was 0.045 with 95% CI ranging from 0.008 to 0.088, excluding 0, indicating that the mediating effect of social motivation was primarily driven by the pathway of social motivation for new.

## 4 Discussion

This study explored social withdrawal in CRC survivors with permanent stomas and examined the relationships among stigma, social motivation, and social withdrawal. Based on the “why try” effect model of stigma, a mediation model was constructed to assess the mediating role of social motivation in the relationship between stigma and social withdrawal. The findings revealed that CRC survivors with permanent stomas commonly reported moderate to high levels of social withdrawal, significantly higher than those observed in 185 patients with bipolar disorder (44) and 243 patients with irritable bowel syndrome (45), suggesting a comparably higher level of social withdrawal in this population. Consistent with the hypotheses, social motivation was found to mediate the association between stigma and social withdrawal, with the mediating effect mainly reflected in the pathway related to motivation for forming new social connections.

The findings indicated a positive correlation between social withdrawal and stigma among CRC survivors with permanent stomas, with higher stigma levels linked to greater social withdrawal. To minimize or avoid external discrimination and rejection, prevent potential embarrassment, and mitigate psychological distress, individuals experiencing stigma often adopt avoidance-based coping strategies, such as withdrawing from social interactions or reducing participation in social activities, to achieve immediate relief (14, 46). Avoidance, as an emotion regulation strategy and defense mechanism, can provide temporary alleviation of stress-related negative emotions (e.g., stigma or social anxiety) and may therefore become reinforced through learned behaviors (47). When individuals experience heightened stigma, their primary psychological goal is to evade situations that may intensify these feelings of shame, distance themselves from social evaluations or pressures, and avoid being observed or judged by others. While this strategy may provide short-term relief, it ultimately exacerbates social withdrawal, reduces opportunities for social engagement, and intensifies social withdrawal. Overall, these findings underscore the need for greater attention to the issue of social withdrawal among individuals experiencing stigma. Targeted clinical interventions aimed at reducing the negative impact of stigma may represent a promising avenue to address social withdrawal and promote psychosocial recovery in CRC survivors with permanent stomas.

The findings indicate a negative correlation between social withdrawal and social motivation among CRC survivors with permanent stomas, suggesting that patients with higher social motivation tend to report lower levels of social withdrawal. Social motivation refers to an individual’s intrinsic drive to engage in social interactions, encompassing the desire to establish and maintain interpersonal relationships. Patients with higher social motivation are generally more inclined to participate in social activities, seeking emotional support and developing social networks through interpersonal interactions. In contrast, those with reduced or diminished social motivation often lack the drive to engage in social interactions, which may be associated with avoidance of social situations, limited social engagement, and greater likelihood of reporting social withdrawal behaviors (48). For CRC survivors with permanent stomas, chronic low-grade inflammation associated with cancer, the psychological and physical stress of ostomy, and chronic stressors such as stigma may be linked to altered reward system functioning, which is important for motivation regulation (49). Such alterations may be associated with reduced perceived reward value of social interactions, lower interest in social engagement, and diminished anticipation of social pleasure, which together are correlated with lower social motivation and greater social withdrawal behaviors (49). Overall, these findings highlight the critical role of social motivation in the process of social withdrawal among CRC survivors with permanent stomas. Enhancing social motivation, particularly by fostering intrinsic motivation for social participation, may represent a promising direction for addressing social withdrawal and supporting social adaptation in this population.

Mediation analysis results indicate that social motivation partially mediates the relationship between stigma and social withdrawal. This suggests that higher levels of stigma are linked to lower social motivation, which is in turn related to greater social withdrawal. From a psychosocial perspective, the feelings of shame induced by stigma often diminish the motivation to engage in social interactions, which is often linked to reduced contact and interaction with others. As social motivation weakens, patients may be more inclined to avoid social situations, which is associated with higher levels of social withdrawal behaviors. From a neurobiological perspective (50, 51), stigma has been linked to alterations in brain regions involved in associative fear learning and social motivation regulation (such as the amygdala, insula, anterior cingulate cortex, and hippocampus), often manifesting as abnormal activation patterns in areas like the prefrontal cortex (PFC) and amygdala. Such patterns are associated with social cognitive biases, heightened sensitivity to social threats, and disrupted social motivation. Notably, the mediating effect of social motivation was primarily driven by the dimension of social motivation for new, which might be attributed to fundamental differences between these two aspects. Social motivation for new emphasizes self-expansion, meeting new people, and forming new relationships, thereby reflecting an active drive to engage in new social interactions. In contrast, social motivation for existing focuses on maintaining stable social networks without necessarily encouraging social expansion. For CRC survivors with permanent stomas, although reliance on existing social relationships is important for care and support, this dependence may not necessarily foster broader social participation. Factors such as physical changes and stigma-related distress may further limit efforts to expand social networks or pursue new social connections, which could explain the weaker association between maintenance motivation and social withdrawal (52). By comparison, higher levels of social motivation for new appear to be linked to greater participation in social interactions, the pursuit of new social support and emotional connections, and lower levels of avoidance and withdrawal. Conversely, lower social motivation for new may be correlated with stronger tendencies toward social withdrawal behaviors.

Although the mediating effect of social motivation is not large, it is important to note that social motivation represents only one dimension of a broader construct. Other unassessed components, such as reward motivation, attentional patterns, and potential neuroregulatory mechanisms (20, 30), may also contribute to stigma-related social withdrawal. Therefore, these findings provide preliminary insight into the potential mechanisms underlying the association between stigma and reduced social motivation. This potential mediating pathway implies that interventions targeting social motivation—particularly those fostering patients’ willingness to engage in social interactions—may help alleviate social withdrawal. However, further experimental studies are needed to confirm the effectiveness of such strategies. Overall, approaches that promote developmental motivation and encourage active participation in new social activities may be promising for supporting social engagement (53). For instance, participation in ostomy support groups may help patients maintain social involvement and reduce social withdrawal (54).

To the best of our knowledge, it is the first study that investigate the relationships between stigma, social motivation, and social withdrawal among CRC survivors with permanent stomas. The results verify the mediating roles of social motivation between stigma and social withdrawal, providing evidence for future interventions to improve psychosocial adjustment to the stoma. This study also highlights the importance of addressing the stigma problems for social withdrawal survivors. Moreover, motivation activation is also important in promoting health behavior (55) and preventing the development of social withdrawal among stigma survivors. Therefore, tailored interventions should be implemented to prevent the development of social withdrawal and promote better psychosocial adaptation outcomes among CRC survivors with permanent stomas. Despite these findings, several limitations should be mentioned. First, a cross-sectional design could not ascertain the causality among stigma, social motivation, and social withdrawal. Longitudinal studies are required to establish causality in the future. Second, due to the small and convenient sample size, the generalizability of the results was limited. A more representative sample with a larger multicenter sample size is warranted in future studies. Third, some potential confounding variables, such as social support level, stoma duration, and comorbidities, were not controlled for in this study and may have influenced the observed associations. Future research should incorporate these factors to enhance the robustness and generalizability of the results. Fourth, the stigma construct was assessed using only two items, which may not fully capture its multidimensional nature. Although internal consistency was acceptable, future studies should employ more comprehensive or validated measures of stigma to strengthen the robustness of the findings. Finally, all variables in this study were assessed through self-reported questionnaires, which may be subject to social desirability and recall biases and could contribute to common method bias. Although Harman’s single-factor test was employed to examine this issue, this approach has inherent limitations (56). Future research should address potential method bias using improved measurement strategies or more robust methodological approaches.

## 5 Conclusions

Moderate to high levels of social withdrawal were commonly reported among CRC survivors with permanent stomas. The findings on the mediating role of social motivation provide valuable insight into how stigma contributes to the development of social withdrawal. Therefore, future interventions aimed at preventing social withdrawal in CRC survivors with permanent stomas should focus on tailored motivation activation strategies, particularly by actively fostering social interaction and encouraging new social connections.

## Data Availability

The raw data supporting the conclusions of this article will be made available by the authors, without undue reservation.
